# Extreme weather events and vector-borne diseases: Quantifying the epidemiological effect of Hurricane Julia on malaria transmission in Colombia

**DOI:** 10.1371/journal.pgph.0006936

**Published:** 2026-08-03

**Authors:** Juan David Gutiérrez

**Affiliations:** Universidad de Santander, Facultad de Ciencias Médicas y de la Salud, Instituto de Investigación Masira, Bucaramanga, Colombia; PLOS: Public Library of Science, UNITED STATES OF AMERICA

## Abstract

Extreme climatic events, such as hurricanes, are known drivers of vector-borne disease outbreaks; however, quantifying their causal effect remains challenging due to complex spatio-temporal dynamics and unobserved confounders. This study estimates the causal effect of Hurricane Julia (October 2022) on malaria cases in Colombian municipalities. We employed a Generalized Synthetic Control (GSC) framework using the fect package in R to estimate the Average Treatment Effect on the Treated (ATT) and the Conditional Average Treatment Effect on the Treated (CATT). Data were obtained from the national public health surveillance system (SIVIGILA) for the period 2013–2023, along with several climatic, environmental, and socio-economic sources. Our results indicate that the hurricane did not have a clear effect on the ATT. Nonetheless, it shows an uptick in malaria transmission after the event, especially when considering forest coverage in the CATT. The results suggest that the disruption caused by the hurricane intensified transmission cycles, particularly in regions with pre-existing socio-ecological vulnerabilities. These results reveal the need for climate-resilient public health strategies and the integration of extreme weather events into early warning systems for malaria control.

## 1. Introduction

Malaria is caused by protozoan parasites of the genus *Plasmodium*, with *P. falciparum* and *P. vivax* accounting for the vast majority of human infections worldwide [1,2]. According to the World Malaria Report, an estimated 249 million cases and 608,000 deaths occurred globally in 2022, representing a marginal increase compared with 2021 [3]. The Americas recorded 723,000 confirmed cases in the same year, with 80% concentrated in the Amazonian sub-region, mainly Brazil (38%), Venezuela (24%), and Colombia (11%) [3]. In Colombia, the National Public Health Surveillance System (SIVIGILA) reported 112,116 cases in 2023, of which 63.1% were caused by *P. vivax* and 35.9% by *P. falciparum*; the Pacific coast and Amazon departments (i.e., administrative level 1), such as Chocó, Amazonas, and Guainía continue to exhibit the highest transmission intensities [4].

A previous study suggested that every 1 °C rise in mean temperature above the local optimum shortened the extrinsic incubation period of *Plasmodium* and increased incidence by 8–15%, whereas annual precipitation between 250–500 mm conferred a 72% higher risk in children compared with areas receiving > 500 mm [5]. These relationships are non-linear: moderate warmth accelerates parasite maturation and mosquito gonotrophic cycles, but excessive heat (> 34 °C) reduces vector survival, not only reduces vector survival but also impairs sporogony in surviving mosquitoes, further suppressing transmission intensity [6], while heavy rainfall can flush larval habitats and transiently suppress transmission [5,7].

Extreme climatic events further amplify this complexity; flooding associated with hurricane or El Niño/La Niña episodes creates numerous stagnant pools that extend vector breeding sites and have been linked to 2- to 4-fold post-event increases in clinical malaria in East Africa [7–9]. Conversely, severe droughts may shrink rivers into discontinuous pools that concentrate both vectors and human water contact, occasionally producing equally intense outbreaks [10].

When a hurricane, flood, or drought strikes, health researchers usually compare disease counts before and after the event, or contrast the affected region with a neighboring one. The problem is that these “before-after” or difference-in-difference approaches assume that both places would have followed the same trend if the disaster had never occurred—an assumption that rarely holds when the extreme climate event also changes migration, income, vector-breeding sites, and public-health responses at the same time [11]. The generalized synthetic control (GSC) avoids this trap: it lets the data pick a weighted set of controls/donor regions whose pre-event disease curve closely mirrors that of the affected areas, then uses those controls to forecast what would have happened after the disaster [12].

Hurricane Julia, the fifth hurricane of the 2022 Atlantic season, initiated as a tropical depression on 6 October and reached Category-1 strength with 140 km/h sustained winds while passing only 15 km south of the Colombian island of San Andrés on the night of 8–9 October. Although the eye did not make landfall on the Colombian continental territory, the storm’s circulation extended 240 km from its center, bringing torrential rain to the entire Colombian Caribbean coast [13]. In La Guajira peninsula the rainfall exceeded 120 mm in 24 h, flooded more than 530 houses, and left 4,000 people temporarily isolated [14].

To our understanding, most of the previous research about the effect of hurricanes on malaria has been focused on descriptive studies or on estimating the association between hurricane damage and malaria risk [15,16]. This research seeks to estimate the causal effect of Hurricane Julia on the case counts of malaria in Colombia, using a GSC model to evaluate the variation in malaria cases attributed to the hurricane in the affected municipalities. The findings from this study could guide public health strategies and risk reduction efforts tailored to malaria and other diseases influenced by climate conditions.

## 2. Methods

We developed an observational longitudinal ecological study; our observational units were the Colombian municipalities. Our goal was to estimate the causal effect of Hurricane Julia on the case counts of malaria in the Colombian municipalities affected by the hurricane. The treatment was defined as municipal exposure to anomalously high rainfall during Hurricane Julia, operationalized as a ≥ 25% positive deviation in mean daily rainfall during the hurricane’s occurrence relative to the historical reference period 1991–2021 (excluding 2020, due to the occurrence of Hurricane Delta), with control municipalities defined as those exhibiting a rainfall deviation of ≤15% during the same period.

### 2.1. Ethical approvals

The Universidad de Santander, through its Institutional Bioethics Board, approved the study with Minutes No. 002 of February 13, 2023. We used anonymized data, and all personally identifiable information was removed during the preprocessing data phase. The shared dataset available in the GitHub repository does not contain any potentially identifying participant information. We developed the study to conform to the Strengthening the Reporting of Observational Studies in Epidemiology reporting guidelines.

### 2.2. Data collection

#### 2.2.1. Malaria cases.

We obtained daily data on malaria cases for all Colombian municipalities from January 2013 to December 2023 from the SIVIGILA, on 28 September 2025. SIVIGILA supplied laboratory-confirmed malaria cases; laboratory analyses were developed at the reporting units (municipalities, departments or the National Institute of Health). To avoid information bias, we removed records with inconsistencies, such as discrepancies in municipality of occurrence, notification date, or age. The number of cases excluded based on these criteria was 32,074, corresponding to 4.6% of all reported cases. According to the National Institute of Health, in the Colombian territory, the malaria transmission cycle is not known to occur at altitudes above 1,600 meters, due to the thermal physiology of the vector [17]. For this reason, we excluded from our study the cases reported in municipalities above this threshold. The number of cases excluded by the altitudinal threshold was 115.

#### 2.2.2. Environmental data.

We obtained daily data on temperature and rainfall from January 2013 to December 2023 from the dataset ERA5 of the European Space Agency, which offers a spatial resolution of 0.10 degrees [18]. To perform the spatial matching between the climate rasters and the municipalities, we implemented the extract function from the raster package in R (version 4.0.3) [19]. This function identified the pixels of temperature and rainfall within each municipal polygon, and we estimated the average temperature and rainfall of each municipality for each day.

We obtained annual raster layers of forest cover from 2013 to 2023, based on the International Geosphere-Biosphere Program classification system. The classes included: Evergreen Needleleaf Forests, Evergreen Broadleaf Forests, Deciduous Needleleaf Forests, Deciduous Broadleaf Forests, and Mixed Forests. These were derived from the NASA MCD12Q1 product with a spatial resolution of 500 m [20], and we estimated the percentage of the municipal area with forest coverage, using the function extract as mentioned above. We procured from the Colombian Mining Monitoring (CoMiMo) the raster layer of illegal mining for 2021 (the only available), with a spatial resolution of 30 m [21]. Note that CoMiMo uses remote sensors to identify illegal open-pit mining. We developed spatial matching as mentioned above, and reported the percentage of the municipal area with illegal mining.

To estimate the distance of each municipality to Hurricane Julia, we first obtained the hurricane’s trajectory from the National Oceanic and Atmospheric Administration [22]. Given that Hurricane Julia followed a trajectory nearly parallel to the Equator across the Colombian Caribbean coast, we approximated the hurricane’s position within each Universal Transverse Mercator (UTM) zone by computing the mean latitude of the recorded trajectory points falling within that zone, while retaining the corresponding UTM easting coordinate. For each municipality, we then calculated the Euclidean distance in kilometers between the municipality’s centroid — projected in the corresponding UTM zone — and the hurricane’s mean position within the same UTM zone, using the full planar coordinates (easting and northing) of both points. Note that the use of UTM allows distance calculations with minimal distortion at the spatial scale of the study area and avoids coordinate inconsistencies between zones that would arise if a single planar projection were used across the entire country.

We obtained the minimal altitude of each municipality from the Agustín Codazzi Geographic Institute [23].

#### 2.2.3. Vector co-occurrence.

We obtained the data of co-occurrence of eight malaria vectors present in Colombia (*Anopheles albimanus, An. calderoni, An. darlingi, An. nuneztovari, An. oswaldoi, An. pseudopunctipennis, An. punctimacula, and An. rangeli*) reported by Gutierrez (2025) [24].

To reduce spatial autocorrelation, spatial filtering with the thin function from R’s spThin package [25] was used, setting a thinning parameter of 25 km^2^ and performing 100 repetitions. The environmental inputs included all 19 bioclimatic variables from WorldClim version 2.1 [26], with a spatial resolution of 30 seconds. Variable selection followed an exhaustive approach as suggested by Cobos et al. (2019) [27]. Initially, Gutierrez (2025) computed the correlation matrix among all 19 bioclimatic variables using occurrence points for each vector species. Subsequently, 10 distinct variable subsets were created by systematically eliminating highly correlated variables (r ≥ 0.7) while ensuring ecological representation. For each subset, correlated variable groups were identified, and one representative variable per group was randomly retained to minimize multicollinearity while preserving the diversity of environmental information.

For model calibration, 16 regularization multiplier values (0.1, 0.2, 0.3, 0.4, 0.5, 0.6, 0.7, 0.8, 0.9, 1.0, 2.0, 3.0, 4.0, 5.0, 6.0, and 10.0) and all 31 possible combinations of feature classes (linear = l, quadratic = q, product = p, threshold = t, and hinge = h) were tested. This resulted in 4,960 candidate models per vector (10 variable subsets × 16 regularization multipliers × 31 feature class combinations). Model selection was based on three criteria: (1) statistical significance determined through partial ROC analysis with 500 iterations and 50% bootstrap resampling (models with p-value < 0.05 were deemed significant); (2) omission rates below 5% using test occurrence data; and (3) model parsimony assessed by Akaike’s information criterion corrected for small sample sizes (AICc), where models with ΔAICc ≤ 2 from the best model were considered equally plausible.

For each vector species, Gutierrez (2025) transformed continuous suitability predictions into binary presence-absence maps using the lowest training presence threshold approach with a 5% omission error rate. This threshold represents the minimum suitability value from the logistic output where presence locations were recorded. Then combined these binary distribution maps to quantify potential vector co-occurrence of four or more vector species for each municipality using the raster package in R (version 4.0.3) [19].

#### 2.2.4. Socioeconomic data.

We downloaded the urban dimension index from the Terridata platform [28], which quantifies the degree and structure of urbanization across municipalities, integrating demographic, spatial, and functional characteristics. It allows for distinguishing urban–rural gradients and identifying patterns of population concentration and service accessibility. Note that the urban dimension index corresponds to a unique measure realized in 2016 by the Planning National Department to identify urban–rural patterns along Colombian municipalities.

The most recent measure of the Multidimensional Poverty Index (MPI) was developed in 2018. The MPI quantifies the proportion of households experiencing multi-faceted poverty within each municipality across the country. The index uses six dimensions: access to public services, conditions affecting children and young people, educational accessibility, employment prospects, healthcare availability, and residential standards. We obtained the MPI from the National Department of Statistics [29].

We obtained the annual population of each municipality from the National Department of Statistics [30].

### 2.3. Hurricane-affected municipalities

We defined hurricane-affected municipalities as those that received anomalously high rainfall during the occurrence of Hurricane Julia (i.e., October 6 – 10, 2022). Anomalies were defined as the difference in percentage between mean daily rainfall across the occurrence of Hurricane Julia compared to the historic reference years 1991 – 2021, excluding 2020, because on October 4 – 9, 2020, occurred the Hurricane Delta [31]. For the main analysis, we defined a cutoff value of>= 25% of difference to distinguish hurricane-affected municipalities. Hurricane-unaffected control municipalities corresponded to municipalities with a difference in percentage <= 15%. Municipalities with a difference in percentage between > 15% and < 25% were classified as buffer municipalities, used to make a sharp transition between hurricane-affected and hurricane-unaffected control municipalities, but not included in the matched control (see 2.4 Matching process).

### 2.4. Matching process

We then used the PanelMatch package in R (version 3.1.1) [32] to create matched sets of control municipalities for each hurricane-affected municipality. The matching procedure operated in two stages.

In the first stage, PanelMatch constructed candidate matched sets by enforcing a treatment history constraint (lag = 12): only municipalities that remained continuously untreated during the 12 periods preceding the hurricane were eligible as controls for each affected unit. This requirement ensures that treated and control municipalities share an identical pre-exposure treatment history over a full 12-period window, eliminating control candidates that may have experienced concurrent or prior exposure to the treatment.

In the second stage, we refined each candidate matched set using Mahalanobis distance matching, which ranks candidate controls by their multivariate pre-treatment similarity to the treated unit [33]. The Mahalanobis distance was computed from the next set of matching covariates:

Group 1 — Lagged outcome history (12 variables). The 12 most recent monthly malaria case counts preceding each time point, represented as individual lag variables (cases lag1 through cases lag12).

Group 2 — Pre-treatment trajectory statistics (3 variables). Three summary measures of the pre-treatment malaria dynamics: (i) mean cases pretreatment, the cumulative mean of malaria cases from the start of the panel up to the period immediately preceding the treatment, computed as a running mean; (ii) standard deviation of cases pretreatment, the cumulative mean of squared deviations from the overall municipal mean, serving as a running variance proxy over the same expanding window; and (iii) trend pretreatment, the slope coefficient from an ordinary least squares regression of malaria cases on time estimated over a rolling six-period window, capturing the short-term trajectory of malaria immediately before the hurricane.

Group 3 — Time-varying environmental covariates (3 variables). Mean temperature, total rainfall, and forest coverage, each evaluated at time of the treatment — that is, at the four-week period of hurricane occurrence — consistent with the standard behavior of the PanelMatch package, which incorporates time-varying covariates at the treatment time point.

Group 4 — Time-invariant municipal characteristics (7 variables). Urban dimension index, malaria vector co-occurrence, log-transformed population size, MPI, illegal mining coverage, minimum altitude, and distance from the hurricane trajectory. These characteristics enter the distance matrix directly as fixed municipal attributes.

Given the high dimensionality of the covariate vector, we used the diagonal of the variance-covariance matrix for distance computation, which standardizes each covariate by its own variance while avoiding instability from off-diagonal elements in high-dimensional settings. For each treated municipality, we selected the size.match = 5 control municipalities with the smallest Mahalanobis distances.

Each of the five selected control municipalities within a matched set received equal weight (1/5), consistent with the default uniform weighting scheme of PanelMatch under Mahalanobis refinement. To construct a single set of municipality-level weights for the subsequent effect estimation, we normalized the weights within each matched set and then averaged them across all treated municipalities. Treated municipalities received a weight of 1. Covariate balance after matching was assessed using standardized mean differences, with a threshold of 0.10 (10%) indicating adequate balance [34].

### 2.5. Effect estimation

After creating comparable groups through matching, we estimated the causal effect of hurricane exposure on malaria case counts, as the Average Treatment Effect on the Treated (ATT). For this goal, we aggregated malaria cases into four-week periods, along with the corresponding average values of temperature and rainfall, to smooth over short-term variation. We estimated the ATT for up to 6 four-week periods following the hurricane. For the ATT estimation, we implemented an interactive fixed effects counterfactual estimator [35]. This approach addresses a fundamental limitation of conventional two-way fixed effects models, which assume that unobserved confounders affect all municipalities identically over time [36]. The interactive fixed effects framework relaxes this assumption by allowing unobserved time-varying factors to have heterogeneous impacts across municipalities [37]. This is particularly relevant in our context, where environmental and socioeconomic factors affecting malaria transmission likely vary both temporally and spatially in complex ways that cannot be captured by simple unit and time fixed effects alone.

The interactive fixed effects model decomposes each municipality’s malaria case count into four components: the treatment effect of hurricane exposure, the influence of observed time-varying covariates (temperature, precipitation, and forest cover), municipality-specific baseline factors that remain constant over time, and the interaction between unobserved time-varying common factors and municipality-specific factors. The unobserved factors can be understood as latent spatial and temporal patterns (such as national-level policies, seasonal disease dynamics, or economic conditions) that affect all municipalities but with varying intensity depending on each municipality’s specific characteristics captured by the factors [37].

To estimate the model parameters and the optimal number of latent factors, we employed cross-validation using pre-treatment observations from the affected municipalities as a validation dataset [38]. Specifically, we evaluated models with zero to five latent factors by holding out one pre-treatment period from hurricane-affected municipalities, fitting the interactive fixed effects model to the remaining data (including all control municipalities and the remaining pre-treatment periods of affected municipalities), and calculating the mean squared prediction error for the held-out observations.

The model that minimized this prediction error was selected as the optimal specification [38]. This data-driven approach avoids arbitrary specification choices and reduces the risk of overfitting while ensuring that the model structure identified from control municipalities generalizes well to the treated units before they were exposed to the hurricane.

We specified a two-way fixed effects structure (unit and time fixed effects) in addition to the interactive components, and we selected the number of factors using the mean squared prediction error criterion as recommended for counterfactual estimation in panel settings [35,38].

Statistical inference was conducted using a parametric bootstrap procedure with 2,500 bootstrap iterations. This approach generates artificial datasets under the null hypothesis of no treatment effect by simulating from the estimated interactive fixed effects model fitted to control observations, applies the matching weights previously calculated by PanelMatch as fixed constants, and re-estimates treatment effects for each simulated dataset. The distribution of these bootstrap estimates provides valid confidence intervals and standard errors that reflect the estimation uncertainty in the interactive fixed effects model parameters and the complex dependence structure in panel data [35,38].

Notably, the bootstrap operates exclusively within the interactive fixed effects estimation stage and does not re-execute the matching procedure in each iteration; the variability introduced by the matching process — including the uncertainty in the Mahalanobis distance calculations and the selection of control municipalities — is therefore not propagated into the reported confidence intervals.

We also calculated the root mean squared error in the pre-treatment period to assess the model’s ability to predict malaria case counts before hurricane exposure, which serves as a diagnostic for model specification and the validity of the parallel trends assumption conditional on the interactive fixed effects structure. Note that in our study, a positive ATT value represents the increase in the number of malaria cases caused by the hurricane, while a negative value indicates a reduction in cases due to the hurricane.

Furthermore, we estimated the Conditional Average Treatment Effect on the Treated (CATT) conditioned on environmental factors, particularly temperature, rainfall, and forest coverage. CATT serves to analyze how the effect of Hurricane Julia on malaria case counts varies across different environmental gradients. The CATT was used to estimate heterogeneous treatment effects over temperature, rainfall, and forest coverage, with the 95% CI calculated to quantify the uncertainty in these estimations. CATT estimation allows for isolation of the marginal effect of each environmental variable, illustrating how the effect of the hurricane on malaria cases varies with temperature, rainfall, and forest coverage while controlling for the spatio-temporal structure of the data.

The CATT was derived from the same interactive fixed effects model used to estimate the ATT. Specifically, for each hurricane-affected municipality, the model produces an individual estimate of the hurricane’s effect by comparing the observed malaria case counts against the counterfactual trajectory predicted in the absence of the hurricane. These individual effect estimates were then smoothed across the range of each environmental variable using a locally weighted regression (LOWESS), yielding a continuous curve that describes how the magnitude of the hurricane’s effect varies across different environmental conditions. The ATT corresponds to the global average of these individual estimates, and is therefore represented as a reference line in the CATT plots.

### 2.6. Sensitivity analysis

All causal inference methods that compare groups over time rely on a fundamental but untestable assumption: that in the absence of the hurricane, malaria trends in affected and unaffected municipalities would have evolved similarly [39]. This assumption, known as parallel trends, cannot be directly verified because we cannot observe what would have happened in hurricane-affected areas had the hurricane not occurred. To assess how sensitive our ATT estimation is to potential violations of this assumption, we conducted sensitivity analyses that explicitly quantify how much our estimated hurricane effect could be influenced by departures from perfect parallel trends.

We implemented two complementary approaches that ask the question: how large would violations of parallel trends need to be to change our ATT estimation? The first approach, called relative magnitude restriction, uses information from the pre-hurricane period to inform plausible violations after the hurricane [39]. Specifically, if there were any pre-existing differences in malaria trends between hurricane-affected and control municipalities before the hurricane occurred, these pre-hurricane differences provide a benchmark for how large post-hurricane violations might reasonably be [35,39].

The second approach, called smoothness restriction, assumes that factors affecting malaria transmission typically change gradually rather than jumping discontinuously at the exact moment of hurricane exposure [39]. For example, vector populations, human immunity patterns, or healthcare access do not typically exhibit sudden breaks coinciding precisely with natural disasters. This approach explores how much the underlying trend differences between groups can change from one period to the next, with larger values permitting more abrupt changes in trends.

For both sensitivity approaches, we used bootstrap resampling with 2,500 iterations to construct statistically valid confidence sets across the 6 four-week periods post-hurricane. These confidence sets indicate the range of possible hurricane effects that are consistent with the data under each assumption about parallel trends violations. Importantly, if these confidence sets include zero, this indicates that the observed data are insufficient to distinguish a true hurricane effect from no effect [39]. This conservative interpretation helps prevent overconfident causal claims when the statistical evidence is inherently limited.

Furthermore, as a third sensitivity analysis, we re-estimated the ATT with a cutoff value of>= 50% of anomalously high rainfall during the occurrence of Hurricane Julia to distinguish hurricane-affected municipalities. Hurricane-unaffected control municipalities corresponded in this sensitivity analysis to municipalities with a difference in percentage <= 40%. Municipalities with a difference in percentage between > 40% and < 50% were classified as buffer municipalities.

The code and dataset for reproducing the results are available on GitHub: https://github.com/juandavidgutier/hurricane_malaria.

## 3. Results

**Fig 1:**
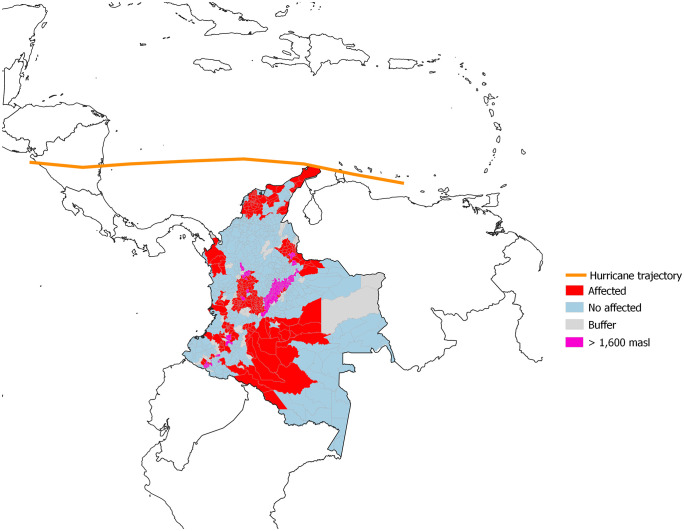
Map of Colombian municipalities presenting the hurricane-affected (red) and hurricane-unaffected control (blue) municipalities. Buffer municipalities that were not included in the matched control are shown in grey. The trajectory of Hurricane Julia is shown with the orange line. Municipalities with more than 1,600 meters above sea level (masl) are represented in purple color. The map was created using QGIS software, with the basemap shapefiles sourced from the Colombian National Geostatistical Framework, (https://www.dane.gov.co/files/geoportal-provisional/SHP_MGN2018_INTGRD_MPIO.zip), and Natural Earth (https://www.naturalearthdata.com/http//www.naturalearthdata.com/download/110m/cultural/ne_110m_admin_0_countries.zip), note that both are openly available resources. The terms of use for the base shapefiles are compatible with CC-BY 4.0 (https://geoportal.dane.gov.co/acerca-del-geoportal/licencia-y-condiciones-de-uso/#gsc.tab=0 and http://www.naturalearthdata.com/about/terms-of-use/).

The number of municipalities included in the study was 877, of which 350 were classified as affected by the hurricane (39.9%) and 527 as unaffected controls (60.1%) (Fig 1). During the study period, 69,584 malaria cases were reported in the affected municipalities (10.4%), whereas 598,180 cases were reported in the control municipalities (89.6%). The top three municipalities with the most cases were Quibdó (Chocó), Tierralta (Córdoba), and Inírida (Guainía), with 61,927, 35,824, and 24,674 cases, respectively.

**Fig 2:**
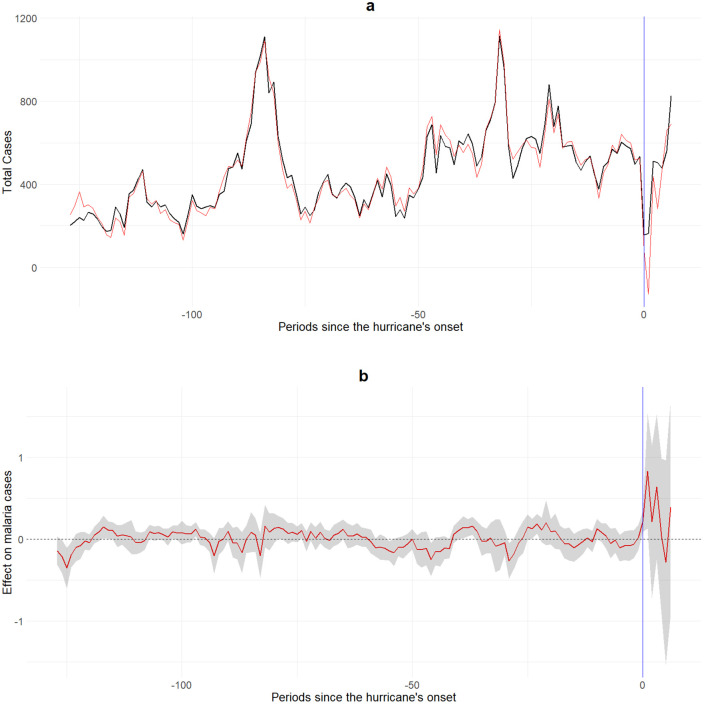
Results of Generalized Synthetic Control (GSC) over time; a) Observed cases (black) across hurricane-affected municipalities compared to cases in the synthetic control (red); b) Effect of Hurricane Julia on malaria cases (red), with corresponding 95% CI (grey band), the horizontal dashed black line indicates no effect. In both panels, the periods pre-hurricane correspond to negative values in the X-axis, and the periods post-hurricane correspond to positive values. The vertical blue line corresponds to the occurrence of Hurricane Julia.

According to the root mean square error (rmse) metric, the GSC model predicted cases well across hurricane-affected municipalities in the pre-hurricane period (rmse = 3.96) (Fig 2a). This means that the typical magnitude of the prediction error, penalizing larger-scale deviations, is 3.96 cases. The ATT for the 6 four-week periods post-hurricane was not different from zero, according to the 95% CI (ATT = 0.030, 95% CI = -0.51 — 1.12). However, the first period post-hurricane showed an ATT different from zero, indicating an average effect of the hurricane, with an average increase of 0.83 malaria cases in the affected municipalities (Fig 2b and Table 1).

**Table 1:** Average Treatment Effect on the Treated (ATT) of Hurricane Julia on malaria cases over the 6 four-week periods across hurricane-affected municipalities.

Period post-hurricane	Dates	ATT	95% CI	p-value
1*	Oct 17 - Nov 13	0.83	0.13–1.51	0.02
2	Nov 14 - Dec 11	0.21	−0.72–1.15	0.66
3	Dec 12 - Jan 8	0.64	−0.25–1.52	0.16
4	Jan 9 - Feb 5	0.03	−0.91–0.98	0.94
5	Feb 6 - Mar 5	−0.28	−1.53–0.96	0.66
6	Mar 6 - Apr 2	0.39	−0.92–1.69	0.56

* = periods post-hurricane with ATT different from zero.

We developed a set of diagnostic tests for the GSC analysis. These tests assess whether our counterfactual model successfully reproduces the malaria trends in the affected municipalities prior to the occurrence of the hurricane.

**Fig 3:**
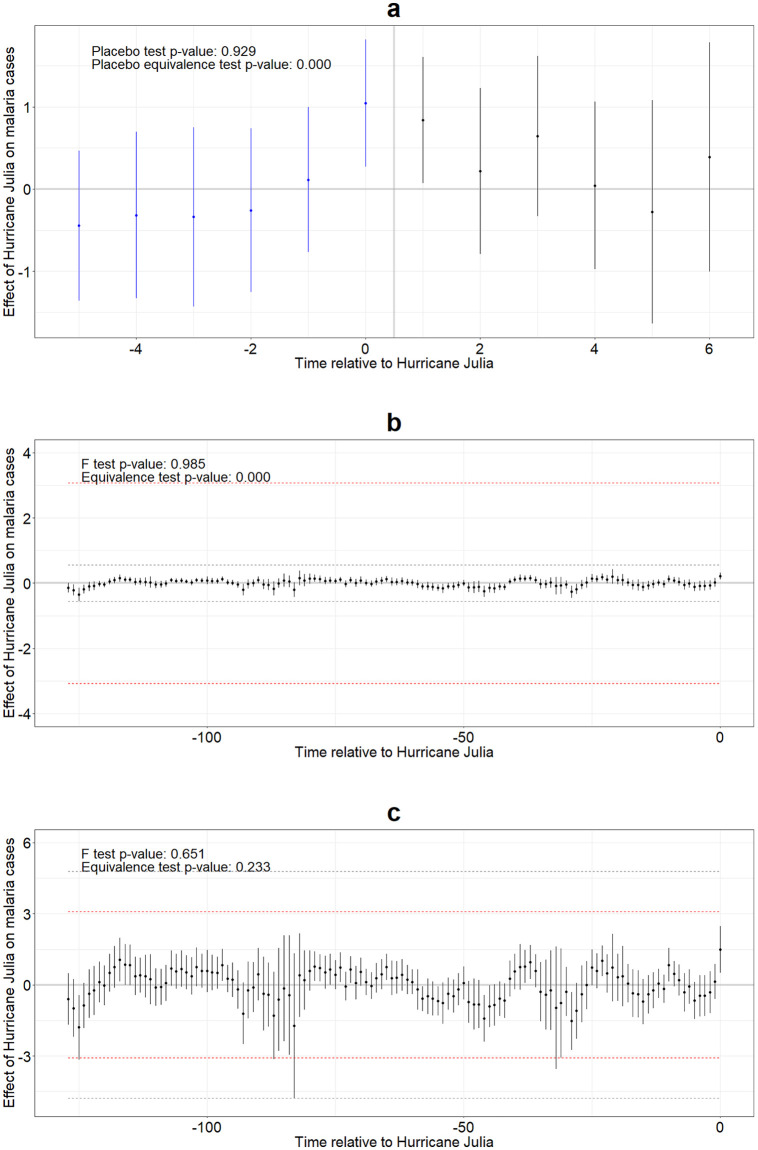
Diagnostic tests implemented to assess whether the counterfactual model successfully reproduces the malaria trends in the affected municipalities before the hurricane: a) placebo test, b) no pre-trend test, c) Leave-One-Out (LOO) test. In all panels, the periods pre-hurricane correspond to negative values in the X-axis, and the periods post-hurricane correspond to positive values. For the no pre-trend test and LOO test, the horizontal red dashed lines define the equivalence bounds representing the maximum tolerable threshold for pre-treatment differences, while the horizontal black dashed lines represent the minimum range that contains the 90% confidence intervals of the actual residuals, indicating a successful fit when the vertical black lines are contained within the horizontal red dashed ones.

We first performed a placebo test to verify the model’s precision immediately before the hurricane. We estimated the treatment effect for a placebo period (i.e., periods -6–0), where no effect should exist. Ideally, the estimated coefficients should be indistinguishable from zero. The results confirm the validity of our control strategy: the estimated effects are centered around zero, and the Placebo test p-value (0.929) indicates that we cannot reject the null hypothesis of no effect. Furthermore, the Equivalence test p-value (< 0.001) confirms that these estimates are statistically equivalent to zero, suggesting no unobserved confounding immediately prior to the hurricane (Fig 3a). Note that while the placebo test checks if the estimated effect is statistically indistinguishable from zero during a specific pre-treatment window, the equivalence test provides a more rigorous validation by statistically proving that the pre-intervention differences between groups are confined within a negligible range of practical insignificance.

To validate the parallel trends assumption over the long term, we examined the trajectory of malaria cases in the affected municipalities versus the estimated counterfactual (i.e., the synthetic control) for the entire pre-hurricane history (Fig 3b). Here, the horizontal red dashed lines represent the pre-defined equivalence bounds—a safety margin within which deviations are considered negligible. The black dashed lines represent the minimum range that encompasses the 90% confidence intervals of the actual data. The fact that the vertical black lines fall entirely within the horizontal red dashed lines visually demonstrates a fit. Statistically, the high F-test p-value (0.985) shows no significant difference between the treated and control groups before the hurricane, while the low Equivalence test p-value (< 0.001) confirms that the pre-treatment difference is practically zero, ensuring the groups were comparable before the disaster.

Additionally, we developed a test for internal fit diagnostics, which verifies the consistency of the latent factors model by reconstructing retained parts of the pre-treatment control data. Specifically, we conducted a Leave-One-Out (LOO) cross-validation test (Fig 3c). Standard pre-trend tests can sometimes be misleading if the same data is used to both fit and test the model. The LOO approach prevents this by estimating the error for each time point using a model trained without that specific time point. Although this is a much stricter stress test resulting in wider confidence intervals, the estimates remain centered around zero. The F-test p-value (0.651) indicates that there is no statistically significant bias in the model’s pre-trend fit, providing evidence that our estimates are not driven by model overfitting. However, the equivalence test p-value of 0.233 in the LOO analysis suggests that the pre-intervention differences are not strictly confined within the narrowest equivalence bounds due to the increased variance of the cross-validation process.

**Fig 4:**
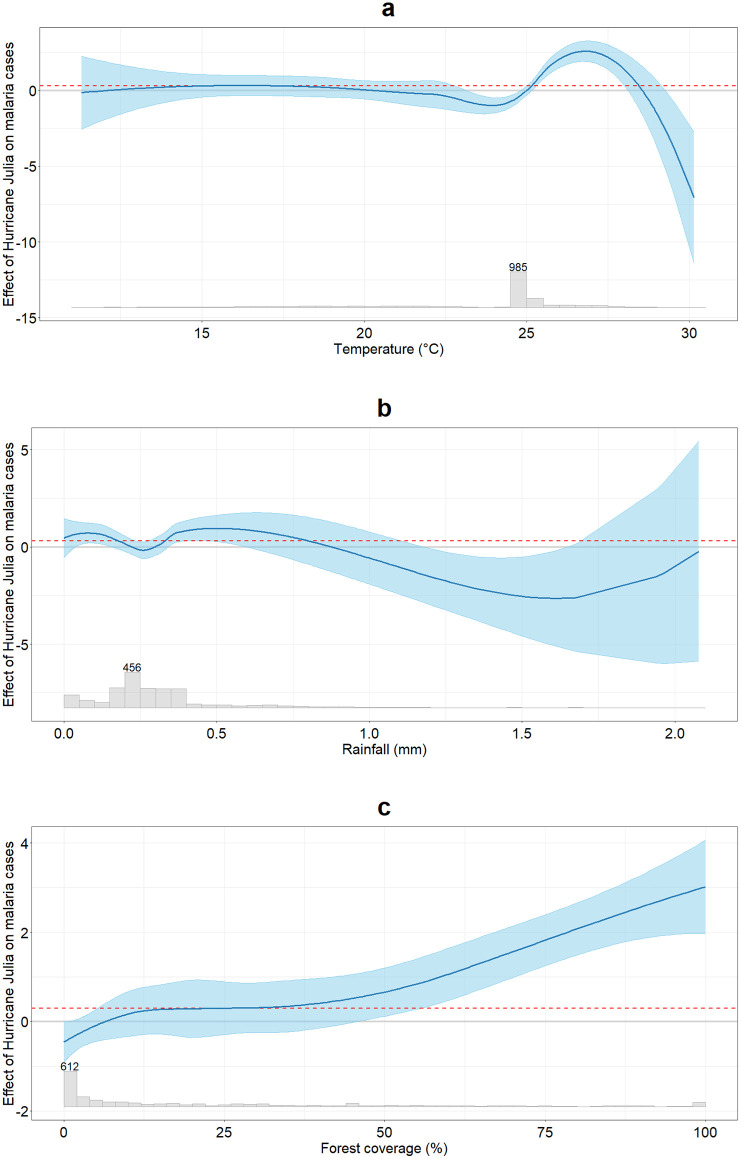
Conditional Average Treatment Effect on the Treated (CATT) conditioned on temperature (a), rainfall (b), and forest coverage (c). The blue line represents the point estimate of the CATT, and the blue band represents the 95% CI. The horizontal gray line represents the null effect, while the dashed red line corresponds to the ATT of the hurricane (i.e., ATT = 0.030). The gray histogram shows the distribution of observations in the affected municipalities.

Fig 4 shows the CATT conditioned on temperature, rainfall, and forest coverage in the municipalities affected by the hurricane. There is a trend towards a larger effect of Hurricane Julia on malaria cases in municipalities with temperatures between 25 and 28 °C (Fig 4a). Meanwhile, municipalities with higher rainfall tend to experience a reduced effect of the hurricane on malaria cases, but this effect is mainly not different from zero (Fig 4b). Greater forest coverage in the municipalities results in a stronger effect of the hurricane on malaria case counts (Fig 4c). Note that the CATT conditioned on forest coverage reaches higher values than the effect of the hurricane alone (ATT = 0.030). This fact suggests an interaction between the effects of Hurricane Julia and forest coverage on the occurrence of malaria cases in the municipalities affected, which highlights the role of forest coverage in the transmission cycle of the disease in Colombia, even during extreme climate events.

The sensitivity tests for relative magnitude restriction and smoothness restriction examine whether the ATT remains valid, even when controlled structural violations of the model assumptions are permitted. This approach, known as honest inference, evaluates whether the estimated effect of the hurricane remains significant even if we allow for minor, realistic deviations in the malaria trends between municipalities.

The two analyses of relative magnitude restriction and smoothness restriction reveal that the relationship between Hurricane Julia and malaria case counts is highly sensitive to these controlled structural violations. Even under the most optimistic scenario—assuming the trends between the affected and control municipalities were perfectly stable—the margins of error (robust confidence sets) included zero. This indicates that the observed fluctuations in malaria cases are not strong enough to be statistically distinguished from natural variability. Therefore, despite our rigorous modeling, the available data does not provide sufficient evidence to confirm a definitive causal link between the hurricane and a change in malaria case counts, i.e., that our estimation of the ATT cannot be considered different from zero, according to the sensitivity tests of relative magnitude restriction and smoothness restriction.

**Fig 5:**
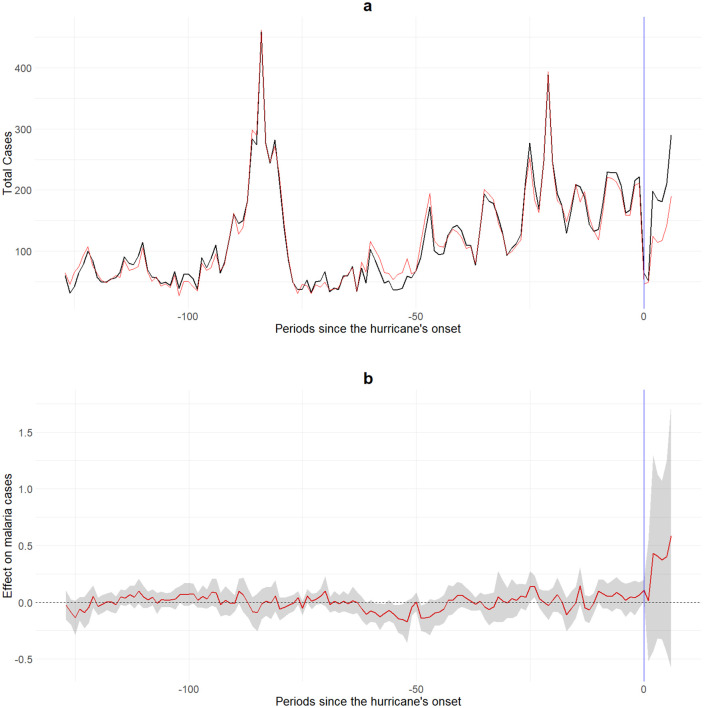
Results of the sensitivity test for a cutoff value of > 50% of anomalously high rainfall during the occurrence of Hurricane Julia to distinguish hurricane-affected municipalities; a) Observed cases (black) across hurricane-affected municipalities compared to cases in the synthetic control (red); b) Effect of the hurricane on malaria cases (red line), the grey band corresponds to the 95% CI. In both panels, the periods pre-hurricane correspond to negative values in the X-axis, and the periods post-hurricane correspond to positive values. The vertical blue line corresponds to the occurrence of Hurricane Julia. The horizontal dashed black line indicates no effect.

When we re-estimated the effect of Hurricane Julia on malaria case counts for the municipalities with > 50% of anomalously high rainfall during the occurrence of the hurricane, we observed a better performance of the GSC model in terms of the rmse metric (rmse = 1.72) (Fig 5a). The average ATT for all post-hurricane periods was not different from zero (ATT = 0.37, 95% CI = -0.23 — 0.96), and neither period post-hurricane has an ATT different from zero (Fig 5b).

## 4. Discussion

Our results cannot provide clear evidence of the ATT of Hurricane Julia on the case counts of malaria in the hurricane-affected municipalities, according to the sensitivity tests of relative magnitude restriction and smoothness restriction, along with the re-estimation of the effect of Hurricane Julia for the municipalities with > 50% of anomalously high rainfall during the occurrence of the hurricane. However, the CATT showed signals of change in the effect of the hurricane, conditioned on environmental variables, particularly in the case of temperature and forest coverage of the affected municipalities.

The CATT, conditioned on temperature, showed a tendency toward a larger effect of the hurricane on malaria cases in municipalities with temperatures between 25 and 28 °C. This finding is consistent with previous research on the thermal physiology of the vector, which predicts optimal malaria transmission at 25 °C and a dramatic decrease in transmission at temperatures above 28 °C [40].

The mechanism behind the steeper increase in post-hurricane malaria observed in heavily forested municipalities remains unclear, but it is likely driven by several mutually reinforcing factors. For example, intact tree cover improves hydrologic pulse, soil infiltration, and slows run-off [41], so rain-water is retained longer in puddles and stream-side pools that serve as persistent *Anopheles* breeding sites.

Colombia harbors three primary malaria vectors — *An. darlingi*, *An. nuneztovari*, and *An. albimanus* — whose bionomics and ecological niches can be relevant to interpreting the CATT results. *Anopheles darlingi* is the dominant vector in the Amazon and humid forests of the Colombian Pacific (particularly Chocó) and is strongly associated with riparian ecosystems and shaded, stable water bodies along forest margins [42]; it exhibits crepuscular and nocturnal biting activity, and its vectorial capacity renders it the most epidemiologically efficient vector in the region [43]. *Anopheles nuneztovari* predominates in the Pacific coast, the Bajo Cauca–Urabá region, and Córdoba, reaching peak abundance at the onset of rainy periods when flooded ponds and swamps expand its larval habitat [42]. *Anopheles albimanus*, the primary coastal vector in the Caribbean thrives in open, sunlit, and brackish water bodies at altitudes below 500 m, with documented natural infection by both *P. falciparum* and *P. vivax* [44].

The pathways through which Hurricane Julia could have modified the vectorial capacity of these species are threefold. First, increased vector abundance: hurricane-induced flooding created extensive networks of stagnant pools and canopy-shaded water bodies that constitute optimal larval habitats for *An. darlingi* and *An. nuneztovari* [42,44], while the recession of floodwaters produces the shallow, sunlit margins favored by *An. albimanus* [44]; this expansion of breeding sites would increase adult mosquito density and consequently modify the daily inoculation rate [45].

Second, increased human–vector contact: the displacement of populations, collapse of housing, destruction of insecticide-treated nets, and disruption of vector control operations following the hurricane would have reduced the physical barriers between humans and vectors, effectively increasing the biting rate [45]. Third, altered vector competence through changes in microclimate: hurricane-induced increases in ambient relative humidity and temperature fluctuations in the 25–28 °C range identified as the CATT optimum in this study are precisely the conditions under which *Anopheles* longevity is maximized and the extrinsic incubation period of *Plasmodium* is shortest, thereby increasing the proportion of infectious mosquitoes in the population [40].

Also note that, some of the country’s forest-rich departments, such as Amazonas, Guainía, and Chocó, also face challenges such as multidimensional poverty, limited access to healthcare, and minimal vector-control coverage [46], making people in these regions more vulnerable to the disease.

Our analysis departs from much of the existing literature by implementing a GSC model, which offers a methodological advancement over traditional epidemiological approaches, such as basic “before-after” comparisons or standard difference-in-differences designs, when evaluating the health impacts of extreme climatic events [11]. Conventional difference-in-differences methods rely on the strict parallel trends assumption, which posits that the treated and control groups would have followed identical trajectories in the absence of the intervention [47]. However, this assumption is frequently violated during climate disasters like hurricanes, which simultaneously alter human patterns, socio-economic stability, and vector breeding dynamics in ways that are not uniform across geography [36]. In contrast, the GSC framework circumvents these limitations by utilizing a data-driven approach to construct a counterfactual from a weighted combination of donor municipalities that best resemble the pre-event characteristics of the affected municipalities [38].

By incorporating latent factors to account for unobserved time-varying confounders, GSC provides a more robust and credible estimate of the causal effect—specifically the ATT—even when dealing with heterogeneous spatial units and complex environmental gradients [35,38]. Furthermore, the integration of the CATT within this framework allows for a nuanced exploration of how environmental effect modifiers, such as local temperature and rainfall or forest cover, amplify or mitigate the epidemiological shock, a level of granularity often unattainable with traditional regression models [35].

Despite the methodological strengths of our study, several limitations must be acknowledged. First, the primary cases data source (SIVIGILA) operates as a passive surveillance mechanism [48]. This structure inherently introduces the risk of information bias, particularly underreporting, as it relies on the systematic notification of cases by healthcare providers rather than active community screening [49]. In the context of Colombia, municipalities with higher MPI scores often face significant structural barriers, including limited diagnostic infrastructure and reduced accessibility to healthcare services. Consequently, the observed case counts of malaria in these marginalized areas may reflect a lower detection and reporting capacity rather than a lower epidemiological burden, potentially attenuating the estimated effect of Hurricane Julia on disease transmission in the most vulnerable regions [50].

The causal analysis was constrained by the technical specifications of the GSC framework as implemented in the fect package in R. While GSC is capable for estimating the ATT by accounting for latent factors and time-varying confounders, the current version of the algorithm does not natively support the inclusion of time-invariant covariates for the estimation of the CATT [35]

This limitation prevented the direct estimation of how fixed structural characteristics, such as baseline poverty or urban dimension, moderate the impact of the hurricane at the municipal level. Although latent factors captured by the model may absorb some of this heterogeneity, the inability to explicitly model these static interactions restricts a deeper interpretation of the socio-economic determinants that could modify the magnitude of the post-disaster epidemiological effect.

A methodological consideration in the present study concerns the operationalization of the outcome variable. The models were estimated using raw case counts rather than population-adjusted rates, a distinction that is epidemiologically relevant given that municipalities vary substantially in population size across the study area. Absolute case counts may therefore reflect demographic heterogeneity rather than true differences in transmission intensity. Whereas the matching procedure partially addresses this issue by including log-transformed population size as a matching covariate — thereby selecting control municipalities with comparable demographic profiles to the hurricane-affected units — this approach does not substitute for the explicit modeling of population-standardized rates. Future applications of counterfactual panel estimators in epidemiological settings would benefit from methodological extensions incorporating count-data likelihoods — analogous to recent developments in Poisson difference-in-differences estimators [51] — which would provide more statistically efficient inference under the overdispersed count distributions typically observed in malaria surveillance data.

A relevant limitation of this study is that the analysis pools malaria cases caused by *P. falciparum* and *P. vivax*, two parasites with different biology, relationships with climatic variables, and seasonality patterns [52]. From a biological standpoint, *P. vivax* has a higher vectorial capacity than *P. falciparum* partly because it completes sporogony at lower temperatures and over a shorter cycle, widening its transmission window into cooler settings and making it less sensitive to some vector control measures [53]. This species-specific thermal niche is directly relevant to the CATT results conditioned on temperature reported here, given that the optimal transmission threshold for *P. vivax* is approximately 2–3 °C lower than that of *P. falciparum* [40].

Moreover, a unique feature of *P. vivax* — absent in *P. falciparum* — is the formation of hypnozoites, dormant liver-stage parasites capable of reactivating weeks to months or even years after the primary infection, producing relapse episodes with renewed blood-stage parasitemia and onward transmission [54]. Critically, hypnozoite reactivation can be triggered by febrile infectious diseases and systemic inflammatory stimuli [55], implying that the physiological stress associated with a hurricane-related disaster could induce relapses of *P. vivax* independently of new vector exposure — a mechanism with no equivalent in *P. falciparum*.

From an ecological perspective, evidence from Colombia and Latin America indicates that precipitation exerts a stronger effect on *P. falciparum* incidence, whereas higher soil temperatures reduce *P. vivax* incidence [56], suggesting that the rainfall-dominated perturbation of Hurricane Julia may have differentially impacted each species. Future studies should stratify analyses by *Plasmodium* species or incorporate the *P. vivax*-to-*P. falciparum* case ratio as an effect modifier within the GSC framework, which would allow detection of whether the heterogeneous treatment effects observed across forest coverage and temperature gradients operate differently according to species composition.

A geometric limitation of the present study concerns the method used to estimate the distance between each municipality and the hurricane trajectory. Given the nearly zonal orientation of Hurricane Julia’s path, we approximated the hurricane’s position within each UTM zone using its mean latitude, and computed planar Euclidean distances between municipal centroids and this mean position within the same UTM zone.

Although this approach is reasonable for a trajectory parallel to the Equator and for municipalities whose centroids lie within the same UTM zone as the hurricane, it does not constitute a fully geodesic distance calculation in the WGS84 ellipsoidal reference system, and the use of mean latitude as a positional proxy introduces a simplification that does not capture the full geometric complexity of the hurricane’s trajectory and extension of the municipalities. Nevertheless, given that distance from the hurricane trajectory was included in the analysis as a matching covariate rather than as the primary exposure variable, and given the relatively compact spatial extent of the hurricane-affected region, this simplification is unlikely to have materially influenced the matching procedure or the causal effect estimates.

## 5. Conclusion

Using a Generalized Synthetic Control framework, this study found no definitive average causal effect of Hurricane Julia on malaria case counts across affected Colombian municipalities; however, the Conditional Average Treatment Effect revealed that the hurricane amplified transmission in municipalities with temperatures between 25–28 °C and higher forest coverage, underscoring the role of local socio-environmental conditions in modulating the epidemiological effect of extreme climatic events and the need for climate-informed early warning systems integrated into national malaria surveillance infrastructure.
