# Supplementary material for: Extreme weather events and vector-borne diseases: Quantifying the epidemiological effect of Hurricane Julia on malaria transmission in Colombia
Source: PLOS Glob Public Health. 2026 Aug 3;6(8):e0006936. doi: 10.1371/journal.pgph.0006936 (PMC13432116; doi:10.1371/journal.pgph.0006936)
Supplement: Attachment — Submitted filename: review report.pdf [file pgph.0006936.s002.pdf]

In this paper, Gutiérrez preformed an analysis of the effects of hurricane Julia on the number of malaria cases in Colombia with a Generalized Synthetic Control framework. The rationale for the study is scientifically sound and I have found the manuscript clear and well written. I have few minor comments below:

- My main issue with this paper is that the analysis pools falciparum and vivax malarias. From my point of view, this is an important limitation as the two parasites have different biology, relationship with climatic variables and seasonality. This should highlighted in the discussion.

- Line 41-42: “A growing body of evidence shows that temperature and precipitation are key climatic drivers of malaria transmission.” Consider rephrasing. I think this has been well identified by early malariologists although there have been only few systematics studies and key knowledge gaps remains.

- Line 47: “excessive heat (> 34 °C) reduces vector survival”. Also impairs parasite sporogony in surviving vectors ?

- Line 94-99: “To avoid information bias […] in municipalities above this threshold.” State what proportion of the original dataset was excluded from the final analysis ?

- Line 193 and throughout the manuscript: “treatment” is misleading consider rewording or give more details this refer to the hurricane at first instance.

- Line 442-445: “This fact suggests an interaction […] a pattern not previously observed”. Although there are some knowledge gaps, there have been previous studies on the role of forest coverage in the transmission cycle malaria in the South Americas. I would suggest to rephrase this statement.

- Line 485-486: “Our results cannot provide clear evidence of the ATT of Hurricane Julia on the case counts of malaria in the hurricane-affected municipalities.” Explain why ?

- Line 498-500: “Second, Colombian forests harbor vectors such as An. darlingi, which prefer stable, shaded water bodies. Their abundance could increase when canopy-covered larval habitats are expanded due to hurricane-induced moderate flooding [41].” Although there have been no direct assessment of the vectors in this study, I think the effects of hurricane on the local vectors in likely a main mechanisms explaining the trends in malaria cases and disserve more details in the discussion. What are the main local vectors, which ones strive in forest and what are their bionomics ? How could the hurricane impact vectorial capacity (e.g. increased vector abundance ? Increased human-vector contact? Increased vector competence because of changes in temperature/humidity conditions ?)

- Line 572-588: The conclusion is rather long. I would suggest to move these three paragraph to the end of discussion and write a one- two-sentence conclusion instead.
