# Supplementary material for: Extreme weather events and vector-borne diseases: Quantifying the epidemiological effect of Hurricane Julia on malaria transmission in Colombia
Source: PLOS Glob Public Health. 2026 Aug 3;6(8):e0006936. doi: 10.1371/journal.pgph.0006936 (PMC13432116; doi:10.1371/journal.pgph.0006936)
Supplement: Attachment — Submitted filename: Response to Reviewers.docx [file pgph.0006936.s001.docx]

Potential Copyright Issues:

a. Figure 1: please (a) provide a direct link to the base layer of the map (i.e., the country or region border shape) and ensure this is also included in the figure legend; and (b) provide a link to the terms of use / license information for the base layer image or shapefile. We cannot publish proprietary or copyrighted maps (e.g. Google Maps, Mapquest) and the terms of use for your map base layer must be compatible with our CC-BY 4.0 license.

R: We accepted the suggestion, and we provide a direct link to the base layer of the maps included in Figure 1, i.e., Countries and Colombian municipalities. Similarly, we provide a link to the terms of use / license information for the shapefiles used in Figure 1 (lines 353 to 364).

Reviewer #1: Major comments

1. If I understand correctly, treatment is defined by rainfall anomaly, and then in the model the authors include precipitation and forest cover as time-varying covariates during the post-treatment period. If the hurricane increases malaria through increased rainfall (a primary mechanism), then controlling for precipitation after treatment can block part of the causal pathway and bias the estimated total effect downward. I think the authors should not control for post-treatment precipitation. They may separate “total effect” from “controlled direct effect” and treat rainfall as mediator explicitly.

R: We sincerely thank the reviewer for this methodologically astute comment. The concern raised reflects a well-grounded principle in causal inference — namely, that controlling for post-treatment variables that lie on the causal pathway between the exposure and the outcome can introduce post-treatment bias and produce estimates of the controlled direct effect rather than the total effect (Pearl, 2009; VanderWeele, 2015). We fully acknowledge the theoretical validity of this concern in the general causal inference framework, and we appreciate the reviewer's diligence in identifying this potential issue.

However, after careful consideration of the specific temporal architecture of our study design — particularly the meteorological duration of the hurricane's direct influence on precipitation relative to our four-week temporal resolution — we respectfully maintain that post-treatment precipitation in our model does not operate as a mediator in the formal causal sense, and that removing it from the model would in fact introduce seasonal confounding bias rather than eliminate post-treatment bias. We elaborate this position below through three complementary arguments.

**Argument 1. Temporal Mismatch Between Hurricane's Direct Precipitation Effect and Post-Treatment Measurement Windows**

The direct meteorological influence of Hurricane Julia on precipitation was confined to the period of its active circulation: October 6–10, 2022 (approximately five days). As a Category 1 hurricane with a rapid translational speed, the organized convective system dissipated within hours to at most one to two days following its passage. Critically, our first post-treatment period begins on October 17 — seven days after the last date of direct hurricane influence — and spans four weeks. The precipitation values averaged over this window therefore reflect predominantly the background seasonal variability governed by large-scale climatic drivers such as the Intertropical Convergence Zone and sea surface temperature, rather than any residual signal attributable to the hurricane. Under these temporal conditions, the causal pathway T → M (hurricane → post-treatment precipitation) is negligible, and the necessary condition for a valid mediation structure is not met at the four-week temporal scale.

**Argument 2. The Treatment Definition Already Operationalizes the Hurricane's Precipitation Signal**

The treatment variable is itself defined by the precipitation anomaly recorded during the hurricane (October 6–10, 2022): municipalities are classified as hurricane-affected if they experienced a ≥ 25% positive anomaly in mean daily rainfall during those dates. The causal signal of the hurricane acting through precipitation is therefore already fully embedded in the treatment assignment mechanism. Precipitation values measured in the subsequent four-week periods represent temporally and causally distinct observations that capture ongoing environmental conditions affecting malaria transmission independently of the hurricane event.

**Argument 3. The CATT Analysis Constitutes Effect Heterogeneity, Not Mediation Analysis**

The reviewer's comment appears to conflate two methodologically distinct estimands: the Controlled Direct Effect (CDE) from mediation analysis, and the CATT from effect heterogeneity analysis. In our study, precipitation is used as an effect modifier to characterize environmental heterogeneity in the hurricane's impact, not as an intermediate variable through which the hurricane exerts its effect — a distinction well-established in the causal inference literature (VanderWeele, 2015; Hernán & Robins, 2020). Removing post-treatment precipitation would not yield the total effect; it would instead fail to account for strong seasonal confounding of malaria by rainfall patterns in tropical Colombia, biasing the ATT estimates in an unpredictable direction.

2. Section 2.2.3 is not clear. Briefly explain how suitability layers were generated in the kuenm framework, which model/s were included and what model inputs were included?

R: We accepted the comment and we have included new paragraphs explaining how suitability layers were generated (lines 132 to 161).

3. PanelMatch typically matches on treatment history for a specified lag and then refines sets via matching orvweighting. It is unclear what the authors mean with “identical temporal conditions”, Mahalanobis distance based on all covariates measured during the 12-month period preceding exposure, and “selected five controls” with inverse-distance weights.

R: We accepted the comment. Note that we operated the matching procedure in two stages. In that sense, we have modified section 2.4 Matching process to clarify the matching method (lines 189 to 233).

4. The authors repeatedly say “incidence” but model “case counts”. If population varies with time, they should either model incidence rates with an offset term, or compute rates explicitly.

R: We agree with the reviewer's comment. Accordingly, we have replaced “incidence” with “case counts” throughout the manuscript. Additionally, we have added a paragraph to the Discussion section acknowledging the limitations of using case counts without an offset term (lines 543 – 555).

5. The authors do parametric bootstrap with 2,500 iterations, which is plausible for GSC-style estimators, but because they do matching, weighting and factor selection, they should clarify whether the bootstrap re-runs matching each iteration or holds matches fixed and only resamples the outcome.

R: We accepted the comment and we have modified the paragraph to clarify this point about the parametric bootstrap (lines 269 to 280).

6. The authors state CATT is “conditioned on temperature, rainfall, forest coverage” and its estimation isolates marginal effect. It is unclear what estimator the authors use for CATT. I think conditioning on rainfall again risks mediator conditioning unless framed explicitly as effect modification of a rainfall-defined treatment.

R: We have accepted the comment and modified the paragraph to explain the estimator used for the CATT (lines 287 – 304). Regarding the concern about conditioning on rainfall risking a mediator: please see our response to the previous identical comment above.

Minor comments

1. Line 108: Thats MODIS land cover - MCD12Q1

R: We have accepted the comment and modified the paragraph to explain which land cover classes from the International Geosphere-Biosphere Program classification system were included in the estimation of the percentage of the municipal area with forest cover (lines 107 - 112).

2. Check lines 110-111: CoMiMo or COMIMO

R: We accepted the comment and we have changed COMIMO to CoMiMo (line 114).

3. Lines 114-119: Consider re-writing this paragraph, latitude alone is not enough for the estimation of the distance from a centroid. Also, were these geodesic distances on WGS84/ or Euclidean in UTM zone?

R: We accept the comment and have modified the paragraph to clarify the procedure for estimating the distance to the hurricane (lines 117 - 129). Additionally, we included a new paragraph in the Discussion section to highlight the limitations of the method (lines 556 - 569).

4. Line 134: Please give the full context of the 2016 reference and provide a reference.

R: We agree with the comment and have revised the sentence to include context for the 2016 reference (lines 166 - 168).

5. Line 158: I beleive the authors just mean time series without cross-sectional added, it is clear from the explanation that follows.

R: Given the modifications made in response to the previous comment regarding the matching process, the term 'cross-sectional' has been removed.

6. Line 173: Should be PanelMatch package, not function. Also check the order of the version specification – as written it appears to be R version and not package version which is referenced.

R: We agree with the comment and have changed "PanelMatch function" to "PanelMatch package" throughout the document. Additionally, we confirm that the version of the PanelMatch package used is 3.1.1.
