# Supplementary material for: Extreme weather events and vector-borne diseases: Quantifying the epidemiological effect of Hurricane Julia on malaria transmission in Colombia
Source: PLOS Glob Public Health. 2026 Aug 3;6(8):e0006936. doi: 10.1371/journal.pgph.0006936 (PMC13432116; doi:10.1371/journal.pgph.0006936)
Supplement: Attachment — Submitted filename: Response_to_Reviewers_auresp_2.docx [file pgph.0006936.s003.docx]

**Comments to the Author**1. If the authors have adequately addressed your comments raised in a previous round of review and you feel that this manuscript is now acceptable for publication, you may indicate that here to bypass the “Comments to the Author” section, enter your conflict of interest statement in the “Confidential to Editor” section, and submit your "Accept" recommendation.

Reviewer #1: All comments have been addressed

Reviewer #2: (No Response)

2. Does this manuscript meet PLOS Global Public Health’s publication criteria? Is the manuscript technically sound, and do the data support the conclusions? The manuscript must describe methodologically and ethically rigorous research with conclusions that are appropriately drawn based on the data presented.

Reviewer #1: Yes

Reviewer #2: Yes

3. Has the statistical analysis been performed appropriately and rigorously?

Reviewer #1: Yes

Reviewer #2: I don't know

4. Have the authors made all data underlying the findings in their manuscript fully available (please refer to the Data Availability Statement at the start of the manuscript PDF file)?The PLOS Data policy requires authors to make all data underlying the findings described in their manuscript fully available without restriction, with rare exception. The data should be provided as part of the manuscript or its supporting information, or deposited to a public repository. For example, in addition to summary statistics, the data points behind means, medians and variance measures should be available. If there are restrictions on publicly sharing data—e.g. participant privacy or use of data from a third party—those must be specified.

Reviewer #1: Yes

Reviewer #2: Yes

5. Is the manuscript presented in an intelligible fashion and written in standard English? PLOS Global Public Health does not copyedit accepted manuscripts, so the language in submitted articles must be clear, correct, and unambiguous. Any typographical or grammatical errors should be corrected at revision, so please note any specific errors here.

Reviewer #1: Yes

Reviewer #2: Yes

6. Review Comments to the AuthorPlease use the space provided to explain your answers to the questions above. You may also include additional comments for the author, including concerns about dual publication, research ethics, or publication ethics. (Please upload your review as an attachment if it exceeds 20,000 characters)

Reviewer #1: In section 2.2.2- line 133-136 of the tracked version (This UTM-based approach ensures that distance...) sounds so AI. Please consider dropping.

R: We accepted the comment and we have rewritten it (lines 132-135).

Reviewer #2: See review report

Reviewer #2

In this paper, Gutiérrez preformed an analysis of the effects of hurricane Julia on the number of malaria cases in Colombia with a Generalized Synthetic Control framework. The rationale for the study is scientifically sound and I have found the manuscript clear and well written. I have few minor comments below:

- My main issue with this paper is that the analysis pools falciparum and vivax malarias. From my point of view, this is an important limitation as the two parasites have different biology, relationship with climatic variables and seasonality. This should highlighted in the discussion.

R: We accepted the comment and we have included new paragraphs in the discussion section to clarify this point (lines 592-614).

- Line 41-42: “A growing body of evidence shows that temperature and precipitation are key climatic drivers of malaria transmission.” Consider rephrasing. I think this has been well identified by early malariologists although there have been only few systematics studies and key knowledge gaps remains.

R: We accepted the comment and we have removed the sentence.

- Line 47: “excessive heat (> 34 °C) reduces vector survival”. Also impairs parasite sporogony in surviving vectors ?

R: We accepted the comment and extended the sentence to include the effect of excessive temperatures on the sporogony of surviving vectors (lines 45-47).

- Line 94-99: “To avoid information bias […] in municipalities above this threshold.” State what proportion of the original dataset was excluded from the final analysis ?

R: We accepted the comment and we have included a new sentence to include the number of excluded cases (lines 99-106).

- Line 193 and throughout the manuscript: “treatment” is misleading consider rewording or give more details this refer to the hurricane at first instance.

R: We appreciate the comment and have included a clear definition of the treatment and control at the beginning of the Methods section (lines 81-86).

- Line 442-445: “This fact suggests an interaction […] a pattern not previously observed”. Although there are some knowledge gaps, there have been previous studies on the role of forest coverage in the transmission cycle malaria in the South Americas. I would suggest to rephrase this statement.

R: We accepted the comment and have rephrased the sentence (lines 449-452).

- Line 485-486: “Our results cannot provide clear evidence of the ATT of Hurricane Julia on the case counts of malaria in the hurricane-affected municipalities.” Explain why ?

R: We accepted the comment and rephrased the sentence to clarify that, according to the sensitivity analyses, the results do not provide clear evidence of an effect of Hurricane Julia (lines 492-498).

- Line 498-500: “Second, Colombian forests harbor vectors such as An. darlingi, which prefer stable, shaded water bodies. Their abundance could increase when canopy-covered larval habitats are expanded due to hurricane-induced moderate flooding [41].” Although there have been no direct assessment of the vectors in this study, I think the effects of hurricane on the local vectors in likely a main mechanisms explaining the trends in malaria cases and disserve more details in the discussion.

R: We appreciate the comment and have included a new paragraph to explain this mechanism (lines 509-519).

What are the main local vectors, which ones strive in forest and what are their bionomics ? How could the hurricane impact vectorial capacity (e.g. increased vector abundance? Increased human-vector contact? Increased vector competence because of changes in temperature/humidity conditions ?)

R: We appreciate the comment and have modified the Discussion section to address these issues (lines 509-535).

- Line 572-588: The conclusion is rather long. I would suggest to move these three paragraph to the end of discussion and write a one- two-sentence conclusion instead.

R: We accepted the comment and modified the Conclusion section (lines 630-637).
